# News Media Coverage of Childcare: How U.S. Local TV News Framed the Problem Before and During the Early Stage of the COVID-19 Pandemic

**DOI:** 10.1007/s10826-023-02573-5

**Published:** 2023-04-17

**Authors:** Margaret E. Tait, Colleen Bogucki, Laura Baum, Erika Franklin Fowler, Jeff Niederdeppe, Sarah E. Gollust

**Affiliations:** 1 Division of Health Policy and Management, University of Minnesota School of Public Health, D305 Mayo Building, MMC 729, 420 Delaware St. SE, Minneapolis, MN USA; 2 86 Ardmore Rd., West Hartford, CT 06119 USA; 3 Wesleyan Media Project, Wesleyan University, 45 Wyllys Ave, Middletown, CT 06459 USA; 4 Department of Government, Wesleyan University, 45 Wyllys Ave, Middletown, CT 06459 USA; 5 Department of Communication, Cornell University, 476 Mann Library Building, Ithaca, NY 14853 USA

**Keywords:** Child care, Public policy, Politics, News media

## Abstract

Early care and education (ECE), or the care young children receive before entering formal schooling, can take multiple forms and is delivered in different settings, such as a center, church, or public school. Federal and state governments regularly fund ECE programs and policies through the Child Care and Development Block Grant Act (CCDBG). Many families, however, face significant challenges in access, cost, and quality of ECE programs, and ECE professionals report substantial challenges in the workplace (e.g., inadequate training) and beyond (e.g., low wages). Policies addressing issues related to ECE were proposed in 2021, but stalled on the U.S. federal policy agenda. In this study, we examine the ECE content of local television news coverage both for its representations of and for its potential influences on ECE policy agendas. We use data from local stations affiliated with the major networks (ABC, NBC, CBS, and FOX) in media markets across the U.S., airing before and during the pandemic. We analyze elements of coverage that could affect public recognition of ECE-related issues, including how problems were framed (e.g., news coverage highlighting scandals or adverse events at ECE facilities) and solutions identified (e.g., public policy). We find that during 2018 and 2019, more coverage highlighted scandalous activity than public policy. The reverse was true, however, during the early period of the pandemic (from mid-March through June of 2020). Researchers and health professionals were seldom included in stories in either sample, and very few stories offered context about the benefits of ECE for health and well-being. These coverage patterns have implications for the public’s understanding of ECE policy and the perceived need for reform. Policymakers, advocates, and researchers looking to advance support for ECE should consider ways to use local television news to present health and policy-relevant information to broad segments of the public.

## Highlights

- Local tv news coverage related to early care and education (ECE) inconsistently highlighted policy, a potential solution to issues of access, affordability, and quality faced by individuals and families.
- In 2018 and 2019, local tv news coverage of ECE frequently highlighted scandalous and adverse events occurring near or with staff from ECE facilities.
- Such coverage – focused on the individual problems associated with ECE - may lead viewers to question the safety and legitimacy of childcare offerings in their community and limit perceptions of systemic issues.
- During the first stage of the COVID-19 pandemic – March through June of 2020 – local tv news coverage of ECE frequently highlighted the policy response to COVID-19 and related provisions for early care and education.
- Researchers and advocates working to advance ECE-related policy should consider the opportunity to partner with local tv news to build awareness of the need for policy reform.

As has been the case with other social safety net issues, the COVID-19 global pandemic has laid bare many faults in the U.S. early care and education (ECE) system. By ECE, we are referring to, “…settings in which children are cared for and taught by people other than their parents or primary caregivers with whom they live,” (Morrissey, 2019). These settings and arrangements, whether a child care center, preschool, or home-based care delivered by relatives or non-relatives, are essential for working parents and caregivers. Despite growing evidence of the relationship between ECE services and participants’ health and well-being across the life course (Almond & Currie, 2010; García & Heckman, 2021; United States Department of Health and Human Services, Administration for Children & Families, 2010), and evidence that these services are particularly impactful among high-risk subgroups (Lee et al., 2021), many families struggle to afford or access ECE. ECE services are also of variable quality, so the services families can afford or access often requires a compromise of quality (in terms of who is caring for a child and what that care includes). These issues of access, affordability, and quality have been exacerbated during the COVID-19 pandemic (Griffin, 2022). ECE operations have been disrupted and individuals and families have had to assume caregiving roles in addition to the challenges they face while working during a pandemic.

Policies related to children and families often receive bipartisan support from voters and policymakers, but recent policy responses to these issues have been increasingly contentious (Haspel, 2021). Heated debates have occurred at the federal level about the extent to which the government should provide support for ECE and consider these investments as infrastructure (Covert, 2021), or whether the cost of care should continue to fall heavily on individuals and families with limited available subsidies (Grose, 2021). Stating it most directly, Wisconsin Senator Ron Johnson described his opposition to government support for childcare in January 2022: “I’ve never really felt it was society’s responsibility to take care of other people’s children” (Linfante, 2022). Despite the bluntness of these most recent federal debates, these conflicts are not new; as Niederdeppe and colleagues (2021) stated: “opposition to accessible and affordable childcare for all—which is often tied to political partisanship and emphasizes themes of family autonomy, parental responsibility, and opposition to government investment in social issues—has emerged in both historical and recent ECE-related policy discussions,” (Niederdeppe et al., 2021).

One reason for why the policy response to ECE in the U.S. has so far been limited could have to do with how the problems ECE addresses are framed in public discourse. For instance, in a synthesis of evidence supporting the association between ECE and child health and well-being, Morrissey and co-authors emphasize a distinction in how ECE investments have been positioned: child care as a support for working families and early education as necessary for child development (Morrissey, 2019). These two alternative framings may have consequences for public support, with the latter potentially seen as more palatable while the former may be less likely to garner support from those who value family autonomy and parental responsibility. In their report detailing efforts to shift and expand the public conversation around early childhood development in Australia, Kendall-Taylor and Lindland made a similar point and highlight the distinction between how experts and the public view childcare: “…while experts see childcare as a site where key developmental processes take place, members of the Australian public have a different mental image of childcare – as a custodial institution where physical safety is the primary concern,” (Kendall-Taylor & Lindland, 2013, p. 7).

It is well known that the media can shape the public’s and policymakers’ agendas and perceptions of policy issues, including for people who do not experience the ECE system directly—but may nonetheless vote or otherwise engage in influencing policy (Baumgartner & Jones, 2009; Kingdon, 1984). Media, through informing, interpreting, and contextualizing content, educate the public and policymakers about issues that could be amenable to policy change (Graber & Dunaway, 2015). Local television news in particular is an important source of health and policy-related related information in the U.S. (Gollust et al., 2019; Pew Research Center, 2020; The Gallup Organization, 2020). While the U.S. public’s trust in media has declined in recent years, local television news remains more trusted. Forty-six percent of respondents surveyed in 2021 reported “a great deal” or “a fair amount of” trust in the information their local TV news station provides related to COVID-19, more trust than was reported in all other sources, including network and cable news or social media (Hamel et al., 2021). Personal connections—in terms of a news outlet covering issues that matter locally or views that journalists are embedded in or a part of a community—influence how people evaluate the media they consume and perceptions of trustworthiness (Gottfried et al., 2020). Local television news offers this personal connection.

Local television news coverage of issues surrounding early childhood thus has a potentially important role in shaping the public’s understanding, but there is only limited research available on media coverage of ECE in the U.S. context. Studies have explored print media coverage in Canada (Albanese et al., 2010), and a single study focused on coverage before and after elections found that Canadian newspapers’ ideological positions were reflected in how they covered issues related to ECE (Rauhala et al., 2012). In one study focused on U.S.-based print media, researchers reviewed newspaper stories from 2000 to 2003 and analyzed the characteristics and themes of pre-kindergarten and early childhood coverage; if and how coverage varied when specific to areas experiencing issues or piloting innovation in ECE; and how newsrooms shape coverage (McAdams & Henry, 2006). They found that newspaper coverage focused on legislation, politics, funding, and facilities, but that the coverage was “inexpert and fairly superficial” (p.45). Another study reviewed both print and television coverage of five children’s issues, including ECE, over a three month study period in spring 2001. These authors found that “…(stories about) child care were consistently overlooked by the print media, and almost invisible on national television newscasts” (Kunkel et al., 2002, p. 8). These U.S.-based studies covered media attention to ECE almost 20 years ago; understanding how media might shape policy agenda-setting today requires an examination of contemporary media coverage. To our knowledge, only one study has examined news coverage of ECE in the context of COVID-19, and this study focused on Canadian news. Wallace and Goodyear-Grant (2020) found that media did not portray ECE as necessary to economic and social recovery from COVID-19, a framing that could advance the issue —one that continues to disproportionately burden women—on policy agendas and motivate systemic change (Wallace & Goodyear-Grant, 2020).

In the present study, we report on a content analysis of local TV news coverage related to ECE during a period pre-pandemic (2018-19) as well as during the early phase of the pandemic (March–May of 2020). Content analysis is the systematic and usually quantitative analysis of the content and meanings of messages (Krippendorff, 2019, p. 51). This method provides researchers with a way to analyze media data for the messages conveyed to the public in news or other public information outlets. Our objectives in this content analysis were to provide evidence of how issues related to ECE were framed to the public in local television news and the extent that public policies were suggested as a solution. We looked at two different windows of time because the latter time period provides a glimpse into how local television news has presented ECE more recently, when the pandemic disrupted existing ECE arrangements and the issues may have appeared more urgent to address, while the earlier news content could have shaped the public’s pre-existing perceptions about this critical system of supports for families.

## Study Data and Methods

This study does not involve human subjects research and institutional review was not required.

### Research Questions

The following questions guided the study design:

(1) How was ECE presented to the public in local television news?

(1a) To what extent were ECE-related issues discussed in the context of scandal-related activity or adverse events?

(1b) Were individuals affected by ECE issues; health care providers and advocates; or political figures included in coverage?

(2) Was public policy discussed in coverage?

### 2018–2019 TV Data

We purchased local TV news broadcasts from all 210 media markets in the U.S. for this study from TVEyes, a commercial search engine for TV coverage. We developed a set of keywords to search closed captioning of local TV news broadcasts to identify a sampling frame of stories related to ECE (see keywords in supplemental appendix 1). We implemented these searches using a system from a commercial data provider, TVEyes. TVEyes enables text-based searching of their database of closed captioning transcripts of broadcasts. We searched broadcasts airing between October 1, 2018 and July 31, 2019, the time period for which we had full broadcast video files available. Using keyword searching to hone in on potentially relevant coding for further analysis has been used in other studies (see Xu et al., 2022) and was implemented to identify potentially relevant news broadcasts for subsequent human coding. Because this approach yielded too many keyword hits (*N* = 62,842) to systematically code, we implemented a constructed week sampling approach to arrive at a manageably sized, probability-based sample. Constructed week sampling is a validated stratified random sampling technique that produces a final sample representative of all days of the week, accounting for the cyclic variation of news content (Luke et al., 2011). Our interest in this study was in analyzing news coverage that aired during time periods of higher viewership (Pew Research Center, 2019) and when we anticipated there may be more substantive coverage of ECE. Thus, our constructed week did not include weekend days and only included “hits” or results from our search terms from news broadcasts aired in early evening (4–7 pm) and late evenings (8–11:59 pm). We limited our sample to stories airing on the four major networks available in most markets – ABC, NBC, CBS, and FOX.

This sampling approach yielded 2522 stories. To refine the sample to content relevant to our research interests, human coders then excluded stories resulting from keyword hits but that were not actually relevant. These included advertisements; teasers that signaled upcoming news stories; stories for animal and adult daycares; and stories about elementary, middle, or high school. In total, we excluded 737 stories and the final analytic sample for the content analysis included 1785 stories. (See supplementary appendix 2 for a visual depicting the sampling frame.).

### 2020 TV Data

To assess ECE-related coverage in 2020 in the early phase of the COVID-19 pandemic, we implemented a similar process of keyword searching of closed captioning followed by sampling strategy. However in 2020, rather than using TVEyes’s browser-based search tool, we were able to bulk download text (and video) data from TVEyes from all local TV news broadcasts of interest and do the keyword searching using our own scripts to identify potentially relevant content. There is no reason to think that the TV Eyes-based search tool and our own scripts would differ in retrieval since we used the same search terms. Unlike in the previous time period (which incorporated all 210 media markets), we purposively sampled 22 media markets for variation in COVID-19 relevant characteristics (including incidence in early 2020, mortality, racial demographics, and political characteristics). Focusing on a smaller sample was an effort to make the best use of the limited resources available for content coding during the early stage of the COVID-19 pandemic, when local TV news coverage of COVID-19 and its impact on individuals and families was extremely extensive. Thus, the 2018-2019 and 2020 data sets are not directly comparable, but we include the 2020 data to provide an update and preliminary assessment of whether key ECE coverage themes might have changed in the onset of the pandemic.

Given the change in discourse around ECE in 2020, the keywords were slightly different from the 2018-2019 effort (See supplementary appendix 1) but were validated for this sample of content. We conducted an iterative and systematic process to validate our search terms that was used in similar studies (Stryker et al., 2006; Xu et al., 2022). As a first step, we conducted keyword searches of broadcasts’ closed captioning for a randomly selected set of dates within our observation period; this time period was not sampled in the content analysis. The goal was to ensure reasonable recall (how well the search phrases call up all relevant content) and precision (the extent that search phrases avoid irrelevant content). This process identified relevant stories with a recall of 71% and a precision of 88%. The keyword searches yielded 2,015 hits total and 651 hits after implementing a similar constructed week approach for March 12 to June 25, 2020. After removing unrelated stories (see supplementary appendix 3 for a visual depicting the sampling frame), we identified a final sample of 172 stories in the 2020 period that referenced early child care and education.

### Content Coding

Based on messages and themes that emerged from watching a pilot set of initial stories (i.e, inductive), combined with a set of themes about ECE policy we intended a priori to track (i.e., deductive), see Research Questions above, we developed a content analysis instrument for use in identifying policy-relevant elements of coverage for the 2018-2019 story set. Stories varied in how substantively they covered issues of ECE, and so the first step in the coding process was to identify whether a story focused on (how we conceptualized substantive content) or mentioned ECE (what we refer to as a story with only a brief reference to ECE, such as a story about legislative activity that lists ECE policy as one of many other activities). A narrower subset of the coding instrument was applied to mention-level stories, given that these stories were less likely to include content of interest.

The full coding instrument included questions intended to identify whether a story highlighted public policy and politics (including whether political disagreements or bipartisan support were identified); the individuals featured, including policymakers (i.e., government or political officials), health care professionals, researchers, and those sharing their experiences with ECE; as well as references to scientific research. Related to political officials, coders were instructed to rely on visual or audible cues provided in stories to identify their party affiliation, save for those we would expect the majority of viewers to know (e.g., Republican former President Donald Trump). We conceptualized individuals sharing their experiences with ECE as exemplars, defined as a real person on the screen who is identifiable or a real person mentioned by name by a reporter or someone talking about that person, and that the person or reporter discusses that individual’s experience with ECE.

During the inductive work to develop the coding instrument, we observed a number of stories that featured scandals or adverse events occurring at or near ECE facilities, such as harm inflicted on a child by a facility staff member, so we added variables to the codebook to capture this. We defined a scandal as “an action or event at or involving a day care center regarded as morally or legally wrong and causing general public outrage… Actions or events could be criminal or potentially criminal activity and occur at or near an early childhood setting”. We defined adverse events as “emergency events caused by a natural or manmade disasters”. The extent to which these events are communicated in local news sheds light on whether ECE-related issues might contribute to negative public perceptions about these environments, or a desire for policy solutions to address them.

For 2020 coverage, our inductive pilot coding indicated that the stories were substantively different in the context of the pandemic, most often reporting on ECE closures and disruptions in the early lockdown. As a result, we had to adapt the coding instrument applied to the 2018-2019 coverage. As with the other instrument, we also implemented coding in the 2020 sample to capture whether the story content conveyed only brief mentions of ECE in passing or a focus on ECE. The stories with substantive mentions of ECE received much of the same coding instrument as focus-level stories, whereas the stories that only mentioned ECE in passing (i.e., a reference to a day care closure that appeared on screen only) were analyzed with a pared down coding instrument. While we added some new variables to capture pandemic themes (i.e., child care closures, reference to essential workers, the “crisis” of child care), we also included some consistent variables from the 2018-2019 coding instrument, including those described above relevant to public policy and politics, scandals and adverse events, sources and exemplars cited, and use of research. We also coded specifically for references to COVID-19; this was not considered an adverse event.

### Content Analysis

Trained coders applied the content analysis instruments on the final sample of stories for the 2018-2019 content (*N* = 1785) and the 2020 content (*N* = 172). A subset of stories (23% for 2018-2019, and 28% for 2020) were double-coded in order to ascertain inter-coder reliability. We calculated Krippendorff’s alpha, a measure of inter-coder reliability, and only those variables with alphas above 0.65 are presented here. We calculated descriptive statistics (frequencies) for key variables. We also explored geographic variation in overall ECE coverage, based on the raw number of closed captioning hits in the 2018-2019 period, to assess the degree to which ECE-related news discourse may have occurred disproportionately in certain media markets within the U.S. All variables are presented in the supplementary materials with definitions of each variable (see supplementary appendix 4).

## Results

### Local TV News Content related to ECE in 2018-2019

**Fig. 1:**
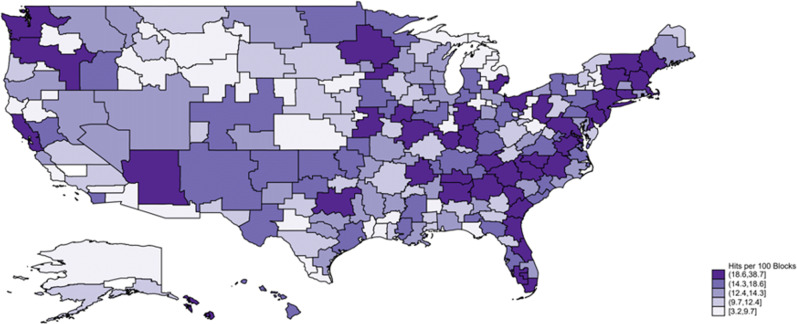
Geographic Variation in Volume of Attention to Early Care and Education in 2018-2019 Local TV News. The figure displays the number of closed caption keyword hits per 100 possible blocks, or broadcasts, available in each media market (see footnote #1)

In 2018-2019, there was a high degree of geographic variation in local TV news coverage of ECE. Fig. 1 displays the variation in attention to ECE on local news broadcasts^1^, displaying that some U.S. media markets saw few to no broadcasts mentioning ECE, while others saw a much higher level of attention to the topic. The highest volume of coverage of ECE in local TV news occurred in the Pacific Northwest, Appalachia region, the Northeast, and the upper Midwest.

Turning now to the analytic sample (*n* = 1785), 37.8% of stories in 2018-2019 focused on ECE (*n* = 952), providing attention to the issues in greater depth, while 33% simply mentioned ECE (*n* = 833) and included only a brief reference. In Table 1, we present information about the stories involving scandal-related content, including among focus- and mention-level stories, as well as the setting and if staff at an ECE facility were involved. Close to twenty percent (19.6%) of all stories referenced scandal-related activity. Scandals were more commonly a part of substantive ECE content: among stories that focused on some aspect of ECE, 31.9% included description of a scandal, but among stories that only mentioned ECE, just 5.5% highlighted a scandal. Adverse events were less commonly described in coverage, appearing in 13% of total stories in the sample; 12.3% of stories that focused on ECE and 13.8% stories that only mentioned ECE. None of the stories in our sample featured both a scandal and an adverse event. Slightly fewer than half of all scandals (42%) described in news content occurred at an ECE facility or involved staff. Close to a third of adverse events (29.3%) took place at an ECE setting or involved facility staff.

**Table 1:** Scandal-related Content in Local TV News Coverage of ECE, 2018-2019 *N* (%)

Stories referencing a scandal	350 (19.6)
Stories referencing an adverse event	232 (13.0)
Stories referencing a scandal or adverse event	582 (32.6)
Stories referencing a scandal and discussion of public policy^a^	12 (4.1)
*Within stories that focus on ECE* | *n* = *952*	
Stories referencing a scandal	304 (31.9)
Stories referencing an adverse event	117 (12.3)
*Within stories that only mention ECE* | *n* = *833*	
Stories referencing a scandal	46 (5.5)
Stories referencing an adverse event	115 (13.8)
*Setting and staff within stories that reference a scandal* | *(n* = *350) or adverse event (n* = *230)*	
Scandals occurring at an ECE facility or involving staff	147 (42.0)
Adverse events occurring at an ECE facility or involving staff	68 (29.3)

^a^The percentage reported is of stories discussing public policy (*n* = 292)

The prevalence of stories mentioning ECE-related public policy in 2018-2019 is reported in Table 2. Less than a fifth (16.4%) of all stories discussed policy. About twenty percent (19.6%) of stories focusing on ECE discussed public policy; this was true of less than fifteen percent (12.6%) of stories mentioning ECE. A small portion (2.2%) of stories mentioned a public budget, and similar to public policy, this code was selected only for budget references relevant to ECE (e.g., a story about the portion of the state education budget going toward early learning). Among stories discussing public policy, slightly more referred to political disagreement than to bipartisanship, but neither of these references to politics were common, occurring in less than 10% of coverage that referenced policy.

**Table 2:** Policy-Relevant Content in Local TV News Coverage of ECE, 2018-2019 *N* (%)

Stories discussing public policy (among total sample)	292 (16.4)
Stories discussing a budget (among total sample)	40 (2.2)
Stories focusing on ECE and discussing public policy	187 (19.6)
Stories mentioning ECE and discussing public policy	105 (12.6)
*Among stories discussing public policy (n* = *292)*	
Stories discussing bipartisanship	20 (6.8)
Stories discussing political disagreement	23 (7.9)

News stories referencing ECE presented a variety of information and source perspectives, as presented in Table 3. Research studies were mentioned in 6.8% of all stories in our 2018-2019 analytic sample, and data visualizations were used in 6.1% of stories. Considering the sources included in coverage, individuals representing advocacy organizations appeared in few stories (2.7%), and even fewer stories included a health care professional (0.5%). Partisan actors were infrequently identified in public policy coverage: Democrats and Republicans were mentioned or pictured in less than five percent of all stories (1.3% for each). Former President Trump was mentioned or pictured in just 6 stories (0.3% of the total sample). More than twenty percent (22%) of stories included an exemplar (an individual mentioned by name or shown on screen sharing their experience with ECE-related issues). Exemplars were a part of close to one third (30.9%) of stories involving a scandal; among these stories with a scandal and an exemplar, the majority (90%) of exemplars were visualized on screen.

**Table 3:** Use of Information, Sources, and Exemplars in Local TV News Coverage of ECE, 2018-2019 *N* (%)

*Use of Research/Evidence*	
Stories referencing a research study	121 (6.8)
Stories including a data visualization	108 (6.1)
*Experts Cited*	
Advocates	48 (2.7)
Health care providers	8 (0.4)
*Reference to partisan sources, among total sample*	
Democrats	23 (1.3)
Republicans	24 (1.3)
*Exemplars*	
Exemplars included in stories	393 (22.0)
Exemplars included in stories involving a scandal ^a^	108 (30.9)
Exemplars visualized in scandal-related coverage^b^	97 (90.0)

^a^The percentage reported is of stories with a scandal (*n* = 350)

^b^The percentage reported is of stories involving a scandal with an exemplar (*n* = 108)

### Local TV News Content of ECE in 2020

After applying relevant exclusions, the final analytic sample of content airing from March 12 until June 25 of 2020 comprised 172 stories. Fewer stories (32.6%) focused on ECE than mentioned it (67.4%). The vast majority (87.8%) mentioned COVID-19 and most of the stories (64%) were in reference to an announcement surrounding the availability of childcare – that is, the opening and/or closing of childcare services in this first phase of the pandemic. Table 4 describes key content features in the 2020 news coverage. In sharp contrast to what was observed in the 2018-2019 sample, less than five percent (2.9%) of stories included a scandal or adverse event. Among all stories, only 2.3% referred to childcare as a crisis and 4.5% referred to childcare as essential. More than one-third of stories (40.1%) discussed public policy and, among these stories, none referred to political agreement or bipartisanship, nor political disagreement. None of the stories in the sample included researchers speaking to ECE-related issues, nor did they refer to “research” or to a “study”. Instead, coverage focused more on individuals speaking to their experience with ECE (i.e., exemplars), which comprised more than one quarter (26.8%) of stories focusing on ECE during this time period.

**Table 4:** Content about ECE in Local TV News, March 12 – June 25, 2020 *N* (%)

*COVID-19-related content*	
Stories referring to COVID-19	151 (87.8)
Stories announcing ECE-related openings or closures	110 (64)
Stories referring to child care as “in crisis”^a^	2 (2.3)
Stories referring to child care as “essential”^a^	4 (4.5)
Stories discussing child care as a benefit to health care or frontline workers ^a^	42 (47.2)
*Scandals and Adverse Events*	
Stories referencing a scandal or adverse event	5 (2.9)
*Policy/Politics*	
Stories discussing public policy	69 (40.1)
Stories discussing private sector policies or plans	11 (6.4)
Stories mentioning bipartisanship	0
Stories mentioning political conflict or disagreement	0
*Sources, Use of Information, and Exemplars*	
Democrats^a^	6 (6.7)
Republicans^a^	2 (2.2)
Stories including a researcher speaking to ECE issues ^b^	0
Stories with references to “research”, “researcher”, or “study” ^b^	0
Stories including a personal exemplar ^b^	15 (26.8)

^a^The percentage reported is of stories focusing on or substantively mentioning ECE (*n* = 89)

^b^The percentage reported is of stories focusing on ECE (*n* = 56)

## Discussion

Local TV news stories in 2018-2019 rarely communicated information to the public that would signal the importance of public policy solutions to the challenges of early care and education in the United States, and the volume of attention to the issue was highly variable across U.S. communities. Less than twenty percent (16.4%) of stories in our 2018-2019 sample discussed ECE in the context of public policy, which is one possible solution to address issues of cost, access, and affordability that many individuals and families have faced for years, and that have been exacerbated by the COVID-19 pandemic. Despite the body of robust research attention to early childhood settings and the impact of a strengthened ECE system on families, communities, and reducing inequality (Anderson et al., 2003; Chaudry et al., 2021), researchers were seldom included in news stories, and only a small number referenced a research study or visualized data.

More stories in 2018-2019 were problem-oriented, rather than policy-oriented, and regularly discussed unsettling activity occurring at or near ECE facilities, such as a crime perpetrated by ECE facility staff, or a natural disaster occurring near a facility. Scandals and adverse events were presented in close to a third of all stories (32.6%), and of the stories involving scandals, close to half (42%) occurred at a facility or involved staff. Of the stories involving a scandal, just 4.1% mentioned public policy, a reference that may have been included as a solution addressing the issue brought to light by the scandal. As a result, the images and stories presented to the public about ECE through local TV news may suggest to the public that the ECE system is rife with “bad actors”. While presentation of scandal-related or otherwise sensational coverage is common in TV news, as others have documented (Grabe et al., 2001), this type of presentation may lead viewers to question the safety and legitimacy of childcare offerings in their community, deter individuals and families from pursuing care necessary for their participation in the workforce, and/or contribute to notions that children should be cared for at home (by mothers). These sentiments comprise prominent counter-arguments to investment in ECE, voiced by the public and policymakers alike (Miller, 2019). While local TV news journalists are responsible for providing and contextualizing information, they are not in the business of demanding reform. Policy advocates and researchers are, and their expertise could compliment local TV news content such that it highlights both the problem and possible solutions, including policy action. Scandal-related ECE content that lacks a connection to public policy as a solution is a missed opportunity to bolster viewers’ interest in policies that could address and reform the very shortcomings of the ECE system leading to such unfortunate events. Further, a focus on specific, sensationally-covered individual episodes at early childhood centers or settings could lead viewers to consider early care and education as an individual problem that families must navigate and make choices on their own to resolve (Iyengar, 1991), not a systematic challenge that affects families and communities across the United States.

In contrast, evidence from our 2020 sample suggests that coverage of ECE in the context of the pandemic focused rarely on scandals or adverse events associated with ECE, and instead included more discussion of public policy. While scandals and adverse events appeared in close to one third of stories in the 2018-19 sample, they were a part of less than five percent (2.9%) of stories in the 2020 sample. Stories highlighting public policy and signaling possible solutions, in contrast, were more prevalent in the 2020 sample and appeared in 40.1% of stories. These references were often to contemporaneous COVID-19-related policy responses (e.g., the CARES Act, and the Families First Coronavirus Response Act) that included resources or regulations for child care centers, as opposed to policy actions specific to ECE. Though more prevalent, public policy-related content did not reference political disagreement or bipartisanship, signals of attention to the politics of the issue (Gollust et al., 2017). The absence of this content in our sample suggests that in 2020, coverage on local television news was more focused on policy than on examining the details of the politics surrounding the issues, politics that became increasingly contentious into 2021 and beyond. Across both samples, coverage often included exemplars, or individuals sharing their experience with ECE (e.g., the difficulty of finding affordable care), in similar proportions. Exemplars were a part of 22.0% of stories in the 2018-19 sample and 26.8% of the 2020 sample. A content analysis of local television news coverage of paid family leave in 2018-19 revealed that just 13.5% of stories included an exemplar (Tait, et. al, 2021). The higher percentage of exemplars in ECE coverage suggests that ECE-related issues could be conducive to the narrative-based frameworks that could be used in ECE policy advocacy (Niederdeppe et al., 2021).

Few other studies have explored news media related to ECE, and this study extends evidence with insights from local television news. Our results suggest that in 2018-2019, local TV news infrequently contextualized ECE in the context of health, social, economic, and accessibility issues. These issues were a part of prominent frames used in recent print media coverage in Canada (Wallace & Goodyear-Grant, 2020) and could motivate support for ECE-related policy reform. Local TV news in the 2020 sample included more discussion of public policy, but with less of the contextualizing factors noted above (e.g., researchers or health professionals as sources) that could bolster support for policy reform or signal societal-level impacts to viewers. References to policy in the 2020 sample were often highlighting state and federal legislative discussions around the COVID-19 pandemic, and not policy specific to address the long-standing problems of access and affordability of ECE that originated prior to the pandemic and persist today.

## Limitations

We offer this evidence while acknowledging the limitations of our research design and to caution against inaccurate conclusions from the results we present. The first limitation is a reminder of our sampling structure: while constructed week sampling offers a probability-based representative sample, it is not the universe of local news coverage that could have discussed ECE during the time periods we examined. We are also limited by the length and scope of the content analysis instrument we developed and successfully implemented, which does not reveal every element of policy-relevant ECE content. Further, the variables we included in the instrument that were intended to capture content about if and how issues of access, quality, affordability, and equity were discussed in the context of ECE did not have sufficient intercoder reliability, so we were unable to draw conclusions about the extent to which these elements of ECE were included in local TV news in either of the two samples. This also presents as a limitation of what we know about policy-related content, since it was difficult for coders to reliably assess the goals of policy (e.g., equity, affordability, etc). Additionally, while we highlight the geographic variation in ECE-related coverage (see Fig. 1), assessing why there was such variation in coverage is beyond the scope of this article and should be a focus of future research.

## Conclusion

Coverage of ECE on local television news in our pre-pandemic (2018-19) sample was less about policy and more about scandal-related activity at or near childcare facilities. Such a prevalence of problem-oriented coverage that lacks context of structural issues (e.g., low wages for childcare staff or limited support for childcare infrastructure) limits the potential for news media to inform the public and policymakers about the issues that can and should be addressed through policy solutions. By contrast, local TV news coverage in our 2020 sample, comprised of stories airing during the early period of the pandemic, included more policy-relevant content and less scandal-related coverage.

Local television news stations face resource constraints that are common among other news media outlets. Plans to disseminate advocacy messages or research evidence should include local TV news producers as an audience. Researchers increasingly connect with journalists on social media platforms and encourage engagement with their work. Targeting local news stations through these efforts may be an effective and efficient way to extend the reach of their expertise to local TV news viewers.

The ECE crisis is not new. It was during a new crisis, though—the COVID-19 pandemic—that local TV news focused ECE-related coverage on policy to support ECE in greater proportion to scandal-related or sensationalized coverage. Early iterations of the Biden administration’s social policy agenda in 2021—the Build Back Better Act—would have allocated significant resources to ECE by way of funds to support free, universal pre-K for all children (Camera, 2021); however, the Build Back Better Act did not pass the Senate, and its successor, the Inflation Reduction Act, did not incorporate ECE provisions (McPherson, 2022). Responding to this legislative failure, President Biden has gone so far as to try to incorporate accessible child care provisions into other, seemingly-unrelated policy, such as a March 2023 Department of Commerce proposal to require computer chip manufacturers to make child care available as a pre-condition for funding (Lopez, 2023). This is a signal of the persistently lacking political will to pass ECE-related legislation at the federal level, as Democrats and Republicans continue to disagree on how to finance increased investments in ECE (Miller, 2022). The lack of robust attention to ECE in local news suggests that the public may not be adequately informed about the challenges of ECE for the health and wellbeing of individuals and families, and that these issues are not consistently on the agenda of pressing matters being discussed.

## Supplementary Information

Supplementary Information

## Footnotes

1 To display the variation in attention to ECE across markets, we converted the number of total raw keyword hits (*N* = 62,842 described above and in Appendix X) to account for the fact that each of the 210 U.S. media markets had a varying number of stations and news broadcasts. We created a measure of “blocks” (or news broadcasts that were possible in each media market) and then calculated the number of hits per 100 possible news broadcast blocks.
