# Supplementary material for: News Media Coverage of Childcare: How U.S. Local TV News Framed the Problem Before and During the Early Stage of the COVID-19 Pandemic
Source: J Child Fam Stud. 2023 Apr 17;32(6):1617–26. doi: 10.1007/s10826-023-02573-5 (PMC10108775; doi:10.1007/s10826-023-02573-5)
Supplement: Supplementary file 1 — Supplementary Information [file 10826_2023_2573_MOESM1_ESM.docx]

**Supplementary Appendix 1: Keywords**

*2018 - 2019*

"childcare" OR "child care" OR "early childhood development" OR "early childhood education" OR headstart OR "head start" OR pre-k OR "pre-kindergarten" OR preschool OR "day care" OR daycare

*2020*

"child care” OR “childcare” OR “child abuse” OR “preschool” or “pre school” OR “child supervision” OR “daycare” OR “day care” OR “babysit” OR “baby sit” OR “homeschool” OR “home school” OR “foster family” OR “foster care” OR “foster child” OR “foster kid” OR “foster system” OR “summer camp” OR “child welfare center” OR “children welfare center" OR (“parents” AND (“finance” OR “income”) AND (“school” OR “class”))

**Supplementary Appendix 2: 2018-19 Sampling Frame**

**
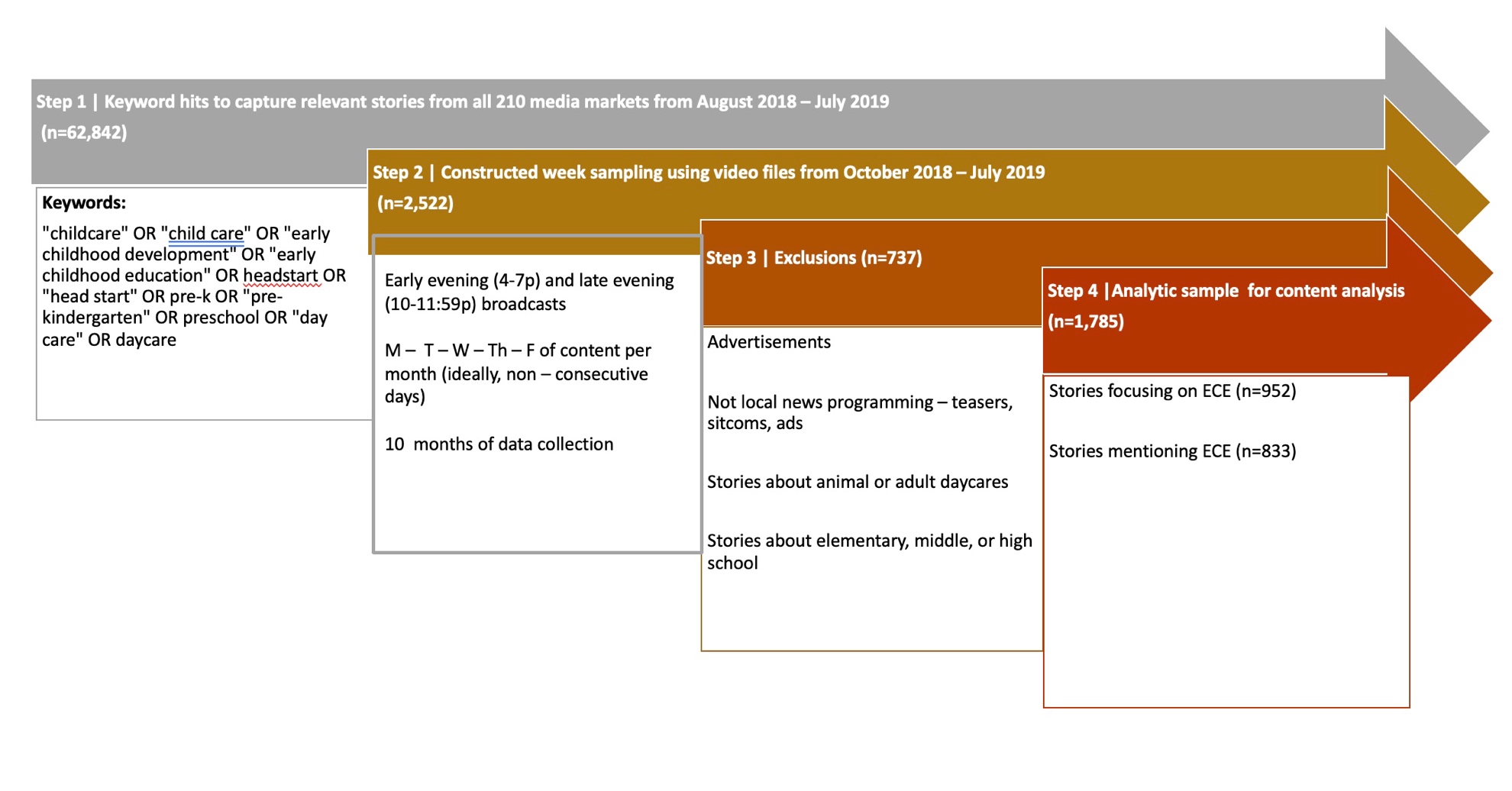
**

**Supplementary Appendix 3: 2020 Sampling Frame**

**
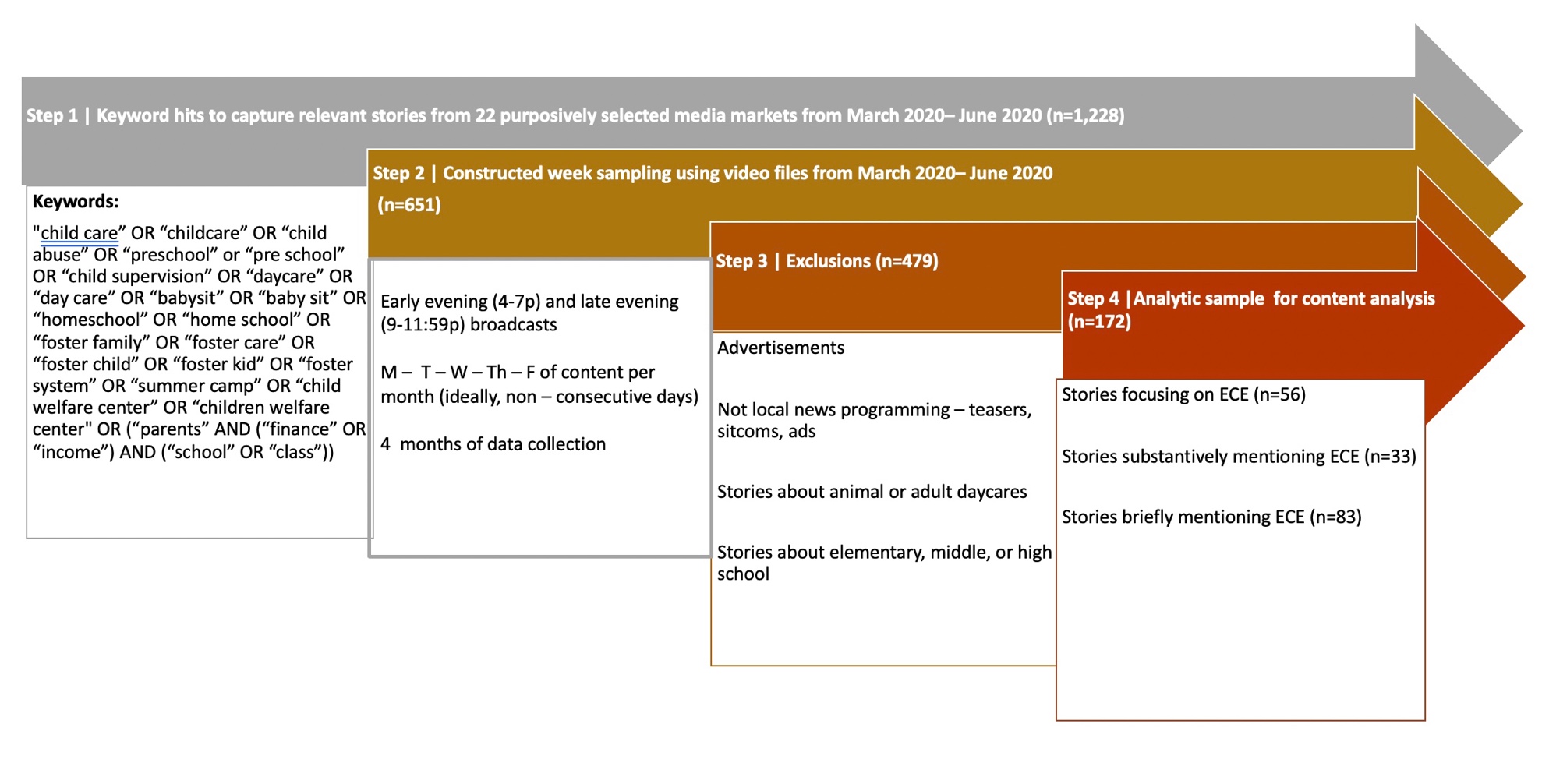
**

**Supplementary Appendix 4: Variables, Definitions, and Krippendorff’s Alpha ICR Values**

| **Variables** | **Definition** | **2018-19 ICR** | **2020 ICR** |
| --- | --- | --- | --- |
| *Values listed as n/a were reported for one sample but not the other.* | | | |
| Early care and education (ECE). | Refers to care and education of children ages 0-5. Includes child care, day care, preschool/pre-k, kindergarten, early care, early education, and summer camp.  Includes only if specifically related to children under 6: foster care, elementary/middle/high school, after school care, K-12 education. | 0.90 | 0.78 |
| Focus on ECE | A story that is primarily about ECE. | 0.90 | 0.78 |
| Mention of ECE | A story that talks about ECE along with other issues, such as a story about what all was proposed or passed during a legislative session. | 0.90 | 0.78 |
| Scandal | An action or event at or involving a day care center (or other ECE setting) regarded as morally or legally wrong and causing general public outrage. Actions or events could be criminal or potentially criminal activity, and occur at or near an early childhood setting. | 0.87 | 1.00 |
| Adverse event | Emergency events caused by natural or manmade disasters. | 0.67 | 1.00 |
| Scandal/adverse event\| Setting is ECE facility | [See above definition of scandal and adverse event.] Examples include a child passing a way at an early learning center. | 0.88 | n/a |
| Scandal/adverse event \| Setting is ECE facility and staff are involved | [See above definition of scandal and adverse event.] Examples include children being harmed or neglected by facility staff. | 0.79 | n/a |
| Public policy | The mention or focus on ECE was in the context of public policy at the local, state, or federal level. Words that signal discussion of public policy include legislation, bill, regulation, lawmaker(s), act, ordinance, executive order, or mandate. | 0.80 | 0.96 |
| Budget | ECE was discussed as part of a budget or proposed budget. Examples include discussion of a Governor’s upcoming budget and inclusion of funding for ECE resources. Mention of ECE as part of local, city, state, or federal budgets also warrant coding here. | 0.80 | n/a |
| Politics \| Bipartisanship | The clip includes discussion of bipartisan efforts, or ideas supported by both Republicans and Democrats, focused on ECE policy. Broad references to bipartisan legislation that is not specific to ECE policy would not be coded here. It is possible for stories to be coded as both bipartisan and disagreement if the story includes examples of both in the same story. | 0.80 | 1.00 |
| Politics \| Political disagreement | Explicit disagreement over the role of government or disagreements over the policy proposed, specific to ECE. This can include disagreements within parties or disagreements between parties. | 0.86 | 1.00 |
| Source \| Republicans | The clip explicitly mentions the term “Republicans” or labels a politician as Republican in mention or in text displayed on screen. This does not include President Trump. The color red is not enough to identify somebody as a Republican. | 0.80 | 0.66 |
| Source \| Democrats | The clip explicitly mentions the term “Democrats” or labels a politician as Democrat in mention or in text displayed on screen. The color blue is not enough to identify somebody as a Democrat. | 0.66 | 0.65 |
| Source \| Former President Trump | (See Wikipedia entry). Includes references to “the president”. | 1.00 | n/a |
| Source \| Individual from advocacy org | An individual advocate or group representing advocates. Interest groups or lobbyists should be coded here. | 0.80 | 1.00 |
| Source \| Health care professional | A doctor, nurse, social worker, or other health professional, or group representing health professionals. | 1.00 | 0.66 |
| Source \| Researcher | An individual researcher or group of researchers.  They may be from an academic institution, a policy group, a think tank, or the government. | n/a | 1.00 |
| Personal exemplar | Clip includes either (a) a real person on the screen who is identifiable OR (b) a real person mentioned by name by a reporter or someone talking about that person; AND (c) the person themselves and/or the reporter discusses that individual’s experience with ECE (e.g. their experience trying to find quality, affordable child care, or their experience providing ECE care) or that individual’s need for ECE policy is described. | 0.70 | 0.99 |
| Personal exemplar visualized | Clip includes a personal exemplar (see above) and that individual is visualized on screen, either as a still image or live action. | 0.70 | n/a |
| Use of information \| Research | The clip explicitly mentions the terms “research”, “researcher”, or “study”. Mentions of “study” should be coded when it used as a noun and not as a verb. | 0.73 | 0.95 |
| Use of information \| Data visualization | The clip includes a visual depiction of a data point, graph, map, or chart. | 0.74 | n/a |
| Reference to COVID-19 | The clip includes explicit reference to “COVID”, “COVID-19”, “coronavirus”, “corona”, “the virus”, and/or “the pandemic/epidemic”. | n/a | 1.00 |
| Child care announcement | Discussion of future activity and not past or present activity related to child care. | n/a | 0.94 |
| Child care is in crisis | The clip explicitly refers to child care as “a crisis” or “in crisis”. | n/a | 1.00 |
| Child care is essential | The clip explicitly refers to child care as “essential” or an essential service. | n/a | 1.00 |
